# The relationship between moral sensitivity and caring behavior among nurses in iran during COVID-19 pandemic

**DOI:** 10.1186/s12912-022-00834-0

**Published:** 2022-03-11

**Authors:** Fatemeh Hajibabaee, Waliu Jawula Salisu, Elham Akhlaghi, Mansoureh Ashghali Farahani, Maryam Mohamadzadeh Nojeh Dehi, Shima Haghani

**Affiliations:** 1 School of Nursing & Midwifery, Tehran University of Medical Sciences(TUMS), Tehran, Iran; 2 Department of Hepatology, Cambridge Liver Unit, Cambridge University Hospitals NHS Foundation Trust. Addenbrooke’s Hospital, Cambridge, England; 3 Nursing Care Research Center, Medical-Surgical Nursing Department, School of Nursing and Midwifery, Iran University of Medical Science, Rashid Yasemi St, Valiasr Ave, Tehran, Iran; 4 Research Development Center, Sina Hospital, Tehran University of Medical Sciences, Tehran, Iran; 5 Biostatistics, Nursing Care Research Center, Iran University of Medical Sciences, Tehran, Iran

**Keywords:** COVID-19, Moral sensitivity, Nursing caring behavior

## Abstract

**Background:**

Caring for patients during a pandemic can be difficult for healthcare workers, the patients themselves, and healthcare systems. Nurses are expected to recognize ethical dilemmas and make sound judgments when confronted with them. Sensitizing nurses to ethical issues strengthen their ability to identify ethical dilemmas and make ethical choices. As a result, this study aimed to determine a relationship between moral sensitivity and caring behavior among nurses during the coronavirus (COVID-19) pandemic.

**Method:**

The current study is a cross-sectional study of 406 nurses who worked in a single hospital during the COVID-19 epidemic. We used a demographic questionnaire and the caring behavior inventory (CBI) tool to collect data online. The data were analyzed using descriptive and correlational statistics.

**Findings:**

Eighty-three point seven percent of participants in this study were female, and 71.9% were married. 47.5% reported caring for a COVID-19 patient for longer than a month; their average work experience was 13.1 years. Additionally, Moral Sensitivity correlated positively with caring behavior and its dimensions (*r* = 0.164, *P* = 0.001). However, a significant and inverse link existed between the dimension "following the rules" and the nurse's caring conduct (*r* = -0.117, *P* = 0.019).

**Conclusion:**

During the pandemic, nurses' moral sensitivity was moderate and significantly connected with their caring behavior. Because nurses encounter numerous obstacles while caring for patients in critical conditions, they require ethical empowerment to perform correctly, as caring behavior improves with increased moral sensitivity.

## Introduction

The emergence of the coronavirus (COVID-19) pandemic has become the biggest challenge for health and economic systems worldwide [1]. According to reports from the World Health Organization (WHO), the number of people infected with the virus as of December 28, 2020, was 79,673,754, with 1,761,381 deaths. In Iran, the first case of COVID-19 was identified in February 2020, and now Iran is the 15^th^ country in terms of the number of cases, with over 1,200,465 positive cases and 45,693 deaths. This unprecedented global situation has caused many nurses worldwide to face ethical issues [2] while caring for their patients [3].

As the largest group of service providers in health care systems, nurses must be morally responsible and accountable for their actions [4, 5]. However, pandemics are fraught with moral distress and psychological challenges, and front-line staff, including nurses, need to be prepared and supported to meet these challenges and uncertainties [6, 7].

It is clear that during this COVID-19 pandemic, clinical work, especially the nursing profession, has faced issues of personal protection and safety. This led to an increased risk of disease transmission among patients, families, and healthcare workers. In addition, the lack of resources and prioritization of care has negatively impacted nurses by increasing work stress and decreasing patient safety [8].

Sensitizing nurses on ethical issues in the professional environment increases their ability to recognize ethical problems, improves the ability to make moral decisions, and leads to the acquisition of problem-solving skills during ethical dilemmas so that clients receive nursing care with confidence and trust (Amiri et al., 2019, Baykara et al., 2015). In this regard, any disruption in nursing ethics observance can affect the most scientific and best nursing care (Dehghani et al., 2013). Moral sensitivity in nurses is essential for decision-making and solving ethical problems [9]. Moral sensitivity is the first of the four components involved in moral decision-making, a combination of individual awareness of moral dimensions such as responsibility, importance to ethical issues, tolerance, and calmness [10, 11].

Ethical sensitivity, on the other hand, is the foundation of ethics in nursing and provides the basis for proper and ethical care of patients [12] and requires the ability to recognize what is right and discuss ethical issues in mind [11]. This internal factor causes people to distinguish between right and wrong and do the right thing. This is related to a person's sense and requires the person's capacity and personal experience to recognize the importance of the moral issue in the situation [13].

Moral sensitivity is a characteristic that enables the nurse to recognize moral challenges, have a correct sensory and intellectual perception of the situation, and finally make decisions based on ethical results [14]. It is an essential condition for moral performance. However, a literature review shows that nurses are sometimes not sensitive enough for various reasons [15].

Among the reasons include nurses' caring role, which significantly impacts healthcare delivery [16]. Besides, the hospital may be unsafe for many and causes tension, which has become more severe in the current pandemic situation than in the past. Nevertheless, nurses' caring behavior can make people feel safe, comfortable and reduce anxiety [17]. The nurse's caring behavior includes all the actions, feelings, cognitions, thoughts, perceptions, gestures, movements, looks, and efforts the client receives care. This caring behavior should be based on ethics [18]. Morse et al. proposed one of the core concepts as care is a moral necessity [19]. Therefore, moral sensitivity, reaction, and attitude development creates a basis for nurses to provide reasonable and ethical care for their patients [12].

Meanwhile, the inability to deal with ethical problems causes several nurses to leave the nursing profession or the desire to change jobs [20]. The waste of labor in this situation does not seem economical because healthcare systems, especially the Iranian nursing community, face a widespread labor shortage [21].

Nurses are the backbone of health care systems worldwide, which is even more evident in the current context because COVID-19 has been associated with uncertainty. However, nurses' role in this pandemic is very prominent [22]. Besides, caring for patients during the COVID-19 pandemic exposed nurses to both physical and emotional challenges such as exposure to disease and fear of infection, disappointing treatment outcomes, high mortality rates, unfamiliar work environment, patients' pessimism due to the psychological impact of others' deaths, nurses' inadequacies in supporting patients, ambiguity in the role of medical staff, lack of experience in infectious disease care, staff stress due to lack of adequate personal protective equipment and staff shortages [23]. Iranian nurses were under much pressure during the COVID-19 pandemic due to a shortage of staff and a high workload. Hiring inexperienced and unskilled staff leads to poor quality, unreliable and error-prone care [24].

Nevertheless, when faced with these issues, the nurse must identify an ethical problem, find possible solutions, and make the appropriate decision. Furthermore, nurses cannot deny the responsibility to respond to patients, so they must provide proper, healthy, and ethical care to all care settings. Hence, given that ethical considerations help spread disease, adopting an ethical approach, and maintaining public trust and confidence [1], a study of the level of Moral Sensitivity and caring behavior of nurses during COVID-19 pandemics seem necessary.

Previous studies in Iran have shown that moral sensitivity is desirable among nurses [25, 26]. However, in the COVID-19 pandemic, nurses' level of moral sensitivity has not been studied. Asadi and colleagues, in their study, showed that the amount of care provided by Iranian nurses in this current pandemic is desirable [27]. Considering the critical role of nurses in delivering high-quality care based on ethics and that the moral sensitivity of nurses during the COVID-19 pandemic is yet to be studied, this study aims to determine the level of Moral Sensitivity of Iranian nurses and its correlation with their caring behavior.

## Materials and methods

### Design

This is a cross-sectional, descriptive-analytical, online study.

### Participants

Four hundred and six nurses across Iran participated in this study. Iran has 31 provinces and about 150,000 nurses working in various hospitals. The sampling method used in this study is snowball sampling. This study's researchers communicated with nurses interested and willing to fill out online questionnaires via Telegram, WhatsApp, and Instagram. In addition, the participants were asked if they knew other nurses who had similar experiences and might be willing to participate. The survey continued until no new participants showed interest. To be included in this study, the participant must be willing to participate and be a qualified nurse working with patients infected with the COVID-19. Participants who declined to participate in the survey or did not complete the online questionnaires as required were excluded.

### Data collection

Data were collected through an online questionnaire from the Porsline platform (a Persian platform used to collect online data) between September 22 to November 30, 2020. In addition, the participants were asked to complete the self-administered questionnaires through mobile phones.

In this study, questionnaires included demographic questions (age, gender, marital status, work experience, department of work, place of employment, history of care for a patient with COVID-19), Lutzen et al.'s Moral Sensitivity Questionnaire. (1994) and the Nurse Care Behavior Inventory (CBI) Questionnaire.

The Moral Sensitivity Questionnaire was developed by Lutzen et al. (1994) [28] and later modified by Comrie [29]. The questionnaire consists of 25 questions that measure nurses' ethical decision-making status during the clinical decision-making process. In this questionnaire, the lowest and highest scores are 0 and 100, respectively. This questionnaire has six dimensions: respect for the client's independence (questions 10, 12, 1), knowledge of how to communicate with the patient (questions 1, 2, 3, 4, 17), professional knowledge (questions 16, 24), the experience of problems and Ethical conflicts (questions 9, 11, 15), the application of moral concepts in moral decisions (questions 6, 8, 14, 18, 20) and the dimension of honesty and benevolence (questions 5, 7, 19, 21, 22, 23, 25). The score of each question is considered by the Likert method as completely agreeing (4), relatively agreeing (3), relatively disagreeing (2), strongly disagreeing (1), and dissenting (0). Therefore, in this study, 0 to 57 will be considered low moral sensitivity, 51 to 75 as moderate moral sensitivity, and 76 to 100 points as high moral sensitivity. The validity of this questionnaire in Iran was examined by Hassanpoor et al. with a reliability of 0.81 [30]. Izadi et al. Also studied the reliability of this questionnaire in 2013, and its internal consistency was calculated with Cronbach's alpha coefficient of 0.8 [18].

The Caring Behavior Inventory (CBI) questionnaire was first designed by Wolf et al., in 1981, with 75 items. After a final revision, it was reduced to 42 items and five subscales in 1998 [31]. The dimensions of this Inventory separately are: “respect for others” (items 1 to 3), “assurance of human presence” (items 1 to 3), “communication and positive trend” (items 1 to 3), “professional knowledge and skills” (items 1 to 3) and “attention to the experience of others” (items 1 to 3). The minimum score is 42, and the maximum is 252. Each item consists of six parts based on the Likert scale and is graded from never (equivalent to one) to always (equal to six). A higher score indicates more appropriate care behaviors [32]. The validity and reliability of this questionnaire were examined in Mahmoodzadeh's study in Iran. Cronbach's alpha coefficient was 87%, and in each of the subscales, between 71 and 87%, with a correlation coefficient of 81% [32, 33]. To confirm the questionnaire’s reliability, the test re-test method was conducted. For this purpose, 20 nurses were selected randomly and completed the questionnaire twice at a 2-week interval. The correlation coefficient between the scores of test–retest scores was 0.9, which showed acceptable reliability. The 20 nurses were excluded from the sample.

### Analysis

The statistical analysis was carried out using descriptive statistics and Pearson correlation coefficient tests for response to the study aim. Data were entered into the statistical package for the social sciences version 21 for windows (SPSS Inc., Chicago, IL, USA).

### Ethical considerations

Study participants entered the study after confirming the explanations in the initial part of the questionnaire, including the study objectives, participants' anonymity, and the optionality of participating in the study in the online questionnaire. This study was approved by the Research and Ethics Committee of Tehran University of Medical Sciences (IR.TUMS.MEDICINE.REC.1399.404). All methods were performed in accordance with the relevant guidelines and regulations.

### Findings

In this study, the majority of the nurses were female (83.7%) and married (71.9%), most (65%) of whom were employed in Tehran. The nurses' mean age was 37.66 ± 8.56 years, and their average work experience was 13.1 ± 8.11 years. In addition, 47.5% stated that they had cared for a patient with COVID-19 for more than a month.

Table (1) shows that the Moral Sensitivity of 78.3% of the nurses was moderate during the COVID-19 pandemic. The mean score of Moral Sensitivity was 62.06 ± 11.49.

The dimensions of nurses' Moral Sensitivity in Table (2) show that “experiencing moral conflict” had an average of 82.48, which was the highest. In contrast, the dimension of “following the rules” had an average of 44.7, the lowest average score among other dimensions.

Table (3) shows that nurses' caring behavior during COVID-19 pandemics in the dimension of “professional knowledge and skills” had an average of 90.01, the highest. On the other hand, the dimension of communication and positive trend” with an average of 78.63 has the lowest average score among different measurements. The mean score of nurses' caring behavior was 84.34 ± 11.4.

**Fig. 1:**
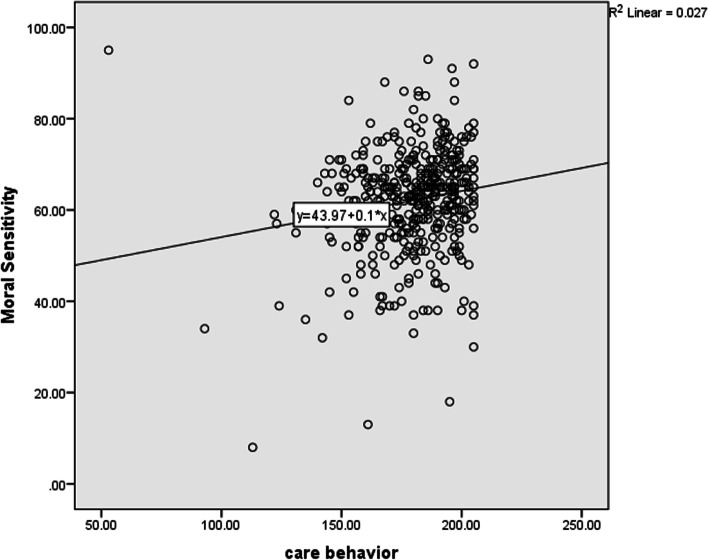
The scatter plot of mental sensitivity with care behavior in Iranian nurses during COVID _19 Pandemic

Moral Sensitivity had a significant positive correlation with caring behavior, and its dimension (*r* = 0.164, *P* = 0.001), i.e., with increasing Moral Sensitivity, caring behavior and its domains also increase (Table 4) and the plot is also reported. However, a meaningful and inverse relationship exists between the “following the rules” dimension and the nurse's caring behavior (*r* = -0.117, *P* = 0.019). With an increase in “following the rules,” caring behavior decreased, but this correlation was positive with other dimensions. The scatter-plot in Fig. 1 shows a relationship between Moral Sensitivity and caring behavior.

**Table 1:** Distribution of frequency, mean and standard deviation of nurses' Moral Sensitivity during COVID-19 pandemic

**Moral Sensitivity**	Percentage	Frequency
Low (50 >)	13.1	53
Medium (75–50)	78.3	318
High (75 <)	8.6	35
Total	100	406
mean ± Standard deviation	62.06 ± 11.49	
Maximum—Minimum	8—95

**Table 2:** Numerical indicators of moral sensitivity and its dimensions in nurses in COVID-19 pandemic

**Moral Sensitivity and dimensions** (0–100)	Maximum—Minimum	mean ± Standard deviation
Modifying autonomy	(10–95)	63.31 ± 16.59
Relational orientation	(0–100)	71.44 ± 18.48
Following the rules	(0–100)	44.7 ± 22.17
Experiencing moral conflict	(0–100)	82.48 ± 22.13
Structuring moral meaning	(0–100)	61.6 ± 16.23
Expressing benevolence	(0–100)	60.59 ± 14.91
Moral Sensitivity	(8–95)	63.06 ± 11.49

**Table 3:** Numerical indicators of nurse care behavior and its dimensions in COVID-19 pandemic

**Nurse care behavior and its dimensions**	Maximum- Minimum	mean ± Standard deviation	Based on (0–100)	
			Maximum- Minimum	mean ± Standard deviation
Respect for others	20–60	52.29 ± 5.74	16.67–100	83.94 ± 11.97
Assurance of human presence	15–60	48.93 ± 5.29	9.09–100	86.22 ± 12.04
Communication and positive trend	9–45	37.31 ± 5.52	0–100	78.63 ± 15.33
Professional knowledge and skills	5–25	23 ± 2.43	0–100	90.01 ± 12.16
Attention to the experience of others	4–20	17.87 ± 2.3	0–100	86.14 ± 14.37
**Nurse care behavior**	53–210	179.32 ± 18.7	7.32–100	84.34 ± 11.4

**Table 4:** Correlation between nurse care behavior and its domains with Moral Sensitivity and its domains in nurses in COVID-19 Pandemic

Nurse care behavior and its dimensions	Moral Sensitivity and dimensions						
	Modifying autonomy	Relational orientation	Following the rules	Experiencing moral conflict	Structuring moral meaning	Expressing benevolence	Moral Sensitivity
Respect for others	*r* = 0.11 *P* = 0.027	*r* = 0.19 *P* = 0.001	*r* = -0.115 *P* = 0.02	*r* = 0.274 *P* = 0.001	*r* = 0.08 *P* = 0.106	*r* = 0.063 *P* = 0.205	*r* = 0.146 *P* = 0.003
Assurance of human presence	*r* = 0.12 *P* = 0.016	*r* = 0.187 *P* = 0.001	*r* = -0.108 *P* = 0.029	*r* = 0.202 *P* = 0.001	*r* = 0.098 *P* = 0.048	*r* = 0.055 *P* = 0.273	*r* = 0.143 *P* = 0.004
Communication and positive trend	*r* = 0.152 *P* = 0.002	*r* = 0.201 *P* = 0.001	*r* = -0.078 *P* = 0.115	*r* = 0.243 *P* = 0.001	*r* = 0.135 *P* = 0.007	*r* = 0.093 *P* = 0.06	*r* = 0.188 *P* = 0.001
Professional knowledge and skills	*r* = 0.055 *P* = 0.27	*r* = 0.039 *P* = 0.435	*r* = -0.086 *P* = 0.083	*r* = 0.189 *P* = 0.001	*r* = 0.014 *P* = 0.779	*r* = 0.06 *P* = 0.226	*r* = 0.02 *P* = 0.689
Attention to the experience of others	*r* = 0.144 *P* = 0.004	*r* = 0.19 *P* = 0.001	*r* = -0.133 *P* = 0.007	*r* = 0.305 *P* = 0.001	*r* = 0.107 *P* = 0.32	*r* = 0.07 *P* = 0.159	*r* = 0.168 *P* = 0.001
**Nurse care behavior**	*r* = 0.137 *P* = 0.006	*r* = 0.199 *P* = 0.001	*r* = -0.117 *P* = 0.019	*r* = 0.267 *P* = 0.001	*r* = 0.107 *P* = 0.031	*r* = 0.063 *P* = 0.204	*r* = 0.164 *P* = 0.001

## Discussion

This study aimed to determine the correlation between moral sensitivity and nurses' caring behavior in the COVID-19 pandemic. This study's findings show a significant positive correlation between moral sensitivity, care behavior, and dimensions. As moral sensitivity increases, so do caring behavior (*r* = 0.164, *P* = 0.001). However, among the dimensions of moral sensitivity, “following the rules” has a significant negative correlation with nurses' caring behavior (*r* = -0.117, *P* = 0.019). This finding contrasts the study results by Izadi and colleagues [18]. In the study of Nasiripour, which investigated the effect of education on promoting moral sensitivity on nurses' care behavior in Ahvaz, a positive relationship was observed between moral sensitivity training and nursing care behavior [34]. in another study by Taylan et al. in Turkey, moral sensitivity was a predictor of caring behaviors [35].

The current study demonstrated that the level of Moral Sensitivity in nurses, based on Moral Sensitivity's mean score, was moderate (62.06 ± 11.49) during the COVID-19 pandemic. The results align with various studies in Iran and some other countries [36–39]. However, other studies have shown a higher level of moral sensitivity of Iranian nurses before the COVID-19 pandemic [40, 41]. Studies show that nurses usually have a sense of responsibility and moral sensitivity, but the central ethical difference lies in adhering to this sensitivity in challenging situations [42]. The COVID-19 pandemic is complex, so hospital officials should identify the factors affecting nurses' moral sensitivity during troubling times, such as taking appropriate steps to develop proper strategies to promote their moral sensitivity.

The present study showed that the highest score of nurses' moral sensitivity in the COVID-19 pandemic was “experiencing moral conflict”, while the lowest score was in the dimension of “following the rules”. The dimension of “following the rules” refers to cases in which decisions are made without the patient's participation. The present results also show that nurses do not involve their patients in treatment and participatory choices. Perhaps nurses involved their patients less in care due to their fears and anxieties and the pandemic's acute state. This finding was consistent with some studies' results [37, 39]. The results of this study are in contrast to the studies of Comrie [29], Lutzen et al. [43], and Amiri et al. [25]. The lowest score in their studies was related to the dimension of experiencing moral conflict. Having moral sensitivity in nurses is vital for decision-making and solving ethical problems [9].

In different dimensions of caring behavior, the average professional knowledge and skills score recorded the highest of 90.01. On the other hand, the dimension of “communication and positive trend” recorded a score of 78.63, being the lowest score.

In the study of Hajinezhad, professional knowledge and skills were more than other dimensions [44, 45]. In this study, it can be concluded that most nurses' emphasis in the current pandemic situation is on behaviors based on knowledge and professional skills. Indicating their ability to perform their professional activities and the importance of this dimension to other dimensions. Studies have also shown that nurses value more for such caring behaviors [15]. Besides, when they do not have enough time and are overworked, nurses may only perform tasks for which they are reprimanded for not performing them. However, the current pandemic has put much workload on nurses, accounting for the low score in some dimensions. The low score in the “communication and positive trend " dimension may also be due to a lack of nursing staff, sufficient time, and fatigue due to forced overtime. Today, the nursing profession is under more pressure than ever, and labor shortages are more significant. As a result, many healthcare workers have contracted COVID-19 disease and must seek treatment by leaving the work environment and remaining quarantined. This study's results are consistent with Wolf and Hajinezhad regarding less "communication and positive trend" compared to other dimensions of care behaviors [45, 46].

### Limitation

We adopted online sampling methods to reach participants due to COVID-19 restrictions and the concern of transmitting or catching the disease. We admit that the online method may reduce the richness of the data as some participants may not provide accurate responses. Also, the approach may have prevented nurses who do not have access to the internet from participating in the study. However, efforts were made to minimize these limitations by widely publishing questionnaires through most social networks where nurses were widely known to be part.

## Conclusion

While the COVID-19 pandemic took the world by surprise, it highlighted numerous challenges. Nurses are critical in containing the pandemic's spread through excellent care and establishing public trust by their continual presence at the bedside of patients. Making ethical choices also significantly impacts nurses' care performance, particularly in the current crisis. The findings of this study indicate that nurses have a modest level of moral sensitivity and that there is a strong association between moral sensitivity and care performance. Compliance with ethical standards in a profession such as nursing can impact an individual's performance when interacting with patients. Nurses must be sensitive to and knowledgeable about ethical issues in their field to respond effectively to clients' rights, autonomy, and evolving ethical matters. Apart from conventional nursing care techniques, they excel in other areas, such as effective communication and patient support, even during pandemics. Future research might look into the aspects that influence care performance and raise nurses' moral sensitivity in critical and pandemic situations and then use that as a model and guidance for building educational programs.

## Data Availability

The datasets used and analyzed during the current study are available from the corresponding author on reasonable request.
